# Some Aegean and Anatolian Hydrobiidae W. Stimpson, 1865: One More Case of Phylogenetically Misleading Morphology

**DOI:** 10.3390/biology15211963

**Published:** 2026-11-01

**Authors:** Aleksandra Jaszczyńska, Deniz Anıl Odabaşı, Caner Aydinli, Artur Osikowski, Sebastian Hofman, Andrzej Falniowski

**Affiliations:** 1 Department of Invertebrate Evolution, Institute of Zoology and Biomedical Research, Jagiellonian University, 30-387 Kraków, Poland; a.jaszczynska@uj.edu.pl; 2 Department of Marine and Inland Water Sciences, Faculty of Marine Science and Technology, Çanakkale Onsekiz Mart University, 17100 Çanakkale, Türkiye; aodabasi@comu.edu.tr; 3 Department of Biology, Faculty of Science, Eskişehir Technical University, 26470 Eskişehir, Türkiye; caydinli@eskisehir.edu.tr; 4 Department of Animal Reproduction, Anatomy and Genomics, University of Agriculture in Krakow, 30-059 Krakow, Poland; artur.osikowski@urk.edu.pl; 5 Department of Comparative Anatomy, Institute of Zoology and Biomedical Research, Jagiellonian University, 30-387 Kraków, Poland; s.hofman@uj.edu.pl; 6 Department of Malacology, Institute of Zoology and Biomedical Research, Jagiellonian University, 30-387 Krakow, Poland

**Keywords:** cytochrome oxidase, integrative taxonomy, morphostatic evolution, molecular phylogeny

## Abstract

The availability of freshly collected *Pseudorientalia natonica* (Küster, 1853) enabled a revision of the putative *Pseudorientalia* Radoman, 1983, species from the Aegean islands of Chios, Samos and Lesvos. It is worth noting that there is no single sequence of ‘real’ *Pseudorientalia* in GenBank. Phylogenetic relationships were studied using cytochrome c oxidase subunit I (COI) sequences, alongside morphological analyses of the shell, radula, female reproductive organs and penes. Molecular data revealed a well-supported *Pseudorientalia* clade that included populations from Chios and Samos. These populations were shown to represent two distinct species that are new to science. Unexpectedly, *Tefennia tefennica* Schütt and Yıldırım, 2003, was nested within this clade. As the observed morphological differences were slight and variable, *Tefennia* is regarded as a junior synonym of *Pseudorientalia*. In contrast, the putative *Pseudorientalia* populations from Lesvos formed a separate, well-supported lineage, being a sister to *Grossuana* Radoman, 1983, and *Daphniola* Radoman, 1973. Despite their close morphological resemblance to *Pseudorientalia*, these populations represent a new genus. A similar pattern was detected in ‘*Pseudorientalia*’ *kanari* D. A. Odabaşı, 2024, which also represents a distinct new genus, and an additional new species was recognised within this lineage. In *Graecoanatolica* Radoman, 1973, marked shell variation was found in a single, molecularly undifferentiated species.

## Simple Summary

Freshwater spring snails of the Hydrobiidae family are often difficult to identify because many species have very similar shells and soft part morphologies. In order to clarify the taxonomy of the putative *Pseudorientalia* species found on the Aegean islands and in Anatolia, we combined DNA sequence data with analyses of shell and anatomical characteristics. Populations from Chios and Samos belong to *Pseudorientalia* but represent two previously undescribed species. We also found that *Tefennia tefennica*, which was previously assigned to a distinct genus, should be included within *Pseudorientalia*. In contrast, populations from Lesvos, despite being morphologically similar to *Pseudorientalia*, belong to a genetically distinct evolutionary lineage representing a new genus. Similarly, *‘Pseudorientalia’ kanari* belongs to a different genus, which also contains a new species. Shells and soft part morphologies can be misleading in hydrobiid snails, so molecular data are essential for resolving evolutionary relationships and defining species and genera. Our study reveals previously unrecognised diversity in Aegean freshwater gastropods and highlights the importance of integrative taxonomy for understanding and conserving this highly diverse yet poorly understood group.

## 1. Introduction

The malacofauna of Anatolia, Greece, and the Aegean islands is rich but still not well studied. This is particularly true of the representatives of the Truncatelloidea J. E. Gray, 1840, the most diverse superfamily of freshwater and brackish water gastropods worldwide. As these snails mostly inhabit springs and subterranean waters, they are especially prone to extinction [1], which makes their study particularly urgent and important. This paper focuses on the genera *Pseudorientalia* Radoman, 1983 (type species: *Paludina natolica* Küster, 1853); *Tefennia* Schütt & Yıldırım, 2003 (type species: *T. tefennica* Schütt & Yıldırım, 2003); *Erosiconcha* Delicado & Gürlek, 2021 (type species: *Pseudamnicola geldiayana* Schütt & Bilgin, 1970); and *Graecoanatolica* Radoman, 1973 (type species: *Hydrobia vegorriticola* Schütt, 1962).

There are four nominal species of *Pseudorientalia*: *P. ceriti* (Gürlek, 2017), *P. intranodosa* (Schütt & Şeşen, 1993), *P. kanari* (Odabaşı, 2024) and *P. naticola* (Küster, 1853). Szarowska et al. [2,3] discovered three formerly unknown species on the Aegean islands: On the islands of Chios, Lesvos and Samos, three formerly unknown species were tentatively classified as belonging to *Pseudorientalia*. Each of these taxa was molecularly distinct, but with no available type species of *Pseudamnicola*, their systematic position remained unclear. Delicado and Gürlek [4] described the genus *Erosiconcha* for *Pseudamnicola geldiayana* (Schütt and Bilgin, 1970) from Akpınar in Denizli, Turkey (the type locality). *Graecoanatolica* is represented by eleven valid species [5], apart from *G. vegorriticola* and *G. macedonica* (Radoman & Stanković, 1979), which are both known only from Anatolia (Turkey). Kebapçı et al. [6] reviewed the Turkish Graecoanatolica.

However, most species of *Pseudorientalia* and *Graecorientalia* have been distinguished based on morphology, often only shell characters, without molecular data. As has already been shown for many truncatelloid snails, it is simply impossible to distinguish these species based on morphology alone [7,8,9,10,11,12,13]. Ecological plasticity, coupled with morphostatic evolution as defined by Davis [14], is a key feature of the wide variability of shells and can characterise genetic modifications and even complete speciation events that are not reflected in morphology. Morphostatic evolution is often the result of non-adaptive radiation, characterised by the rapid proliferation of species without ecological differentiation [15]. This results in a multitude of species that are not necessarily differentiated morphologically. Although traditionally neglected, non-adaptive radiation is not a rare phenomenon in gastropods [13,16,17,18]. GenBank contains no ‘true’ *Pseudorientalia* sequences (only the 45 sequences from the three Aegean islands that have been provisionally assigned to this genus and not to a species). Apart from its type species, *G. vegorriticola*, GenBank contains cytochrome c oxidase subunit I (COI) sequences for the following *Graecoanatolica* species: *G. lacustristurca* Radoman, 1973; *G. macedonica*; *G. pamphylica* (Schütt, 1964); and *G. tenuis* Radoman, 1973.

The availability of freshly collected topotypes of *Pseudorientalia naticola*, the type species of the genus, has provided an opportunity to reassess the systematic position of several morphologically similar hydrobiids from the Aegean islands and Anatolia. The primary objective of this study is therefore to revise the taxonomic status and phylogenetic relationships of putative *Pseudorientalia* populations from Chios, Samos, and Lesvos; reconsider the validity of the genus *Tefennia*; and clarify the systematic position of *Pseudorientalia kanari*. We have also provided new molecular and morphological information on *Erosiconcha* and *Graecoanatolica*. By combining COI-based phylogeny and species-delimitation analyses with shell morphology, geometric morphometrics, radular characters, and reproductive anatomy, we attempted to assess species distinctness. Given the ever-growing body of knowledge on the limitations of mitochondrial DNA-based phylogenies, we also sequenced three nuclear loci where possible.

However, our results clearly illustrate a phenomenon that is not uncommon in minute truncatelloid gastropod systematics above the species level. The anatomy does not offer many characters that are useful for taxonomy. It has been simplified as a result of miniaturisation and unified as a result of necessary adaptations (which have often been gained in parallel and thus represent homoplasies) to life in freshwater habitats (e.g., osmoregulation, internal fertilisation, eggs rich in yolk and embryonic development inside a capsule). While these characteristics are all necessary in freshwater habitats, they are not plesiomorphic for Gastropoda. Thus, anatomical similarities are often the result of parallelism or even convergence and do not necessarily reflect common ancestry. In general, homoplasies are common in Mollusca. The second objective was therefore to evaluate the extent to which morphological similarity reflects phylogenetic relationships in these minute freshwater gastropods. However, there is another side to the coin: in gastropods, the vast diversity of shell form often does not reflect taxonomic diversity, a point we wish to demonstrate in *Graecoanatolica*.

## 2. Materials and Methods

### 2.1. Snail Collection

**Figure 1:**
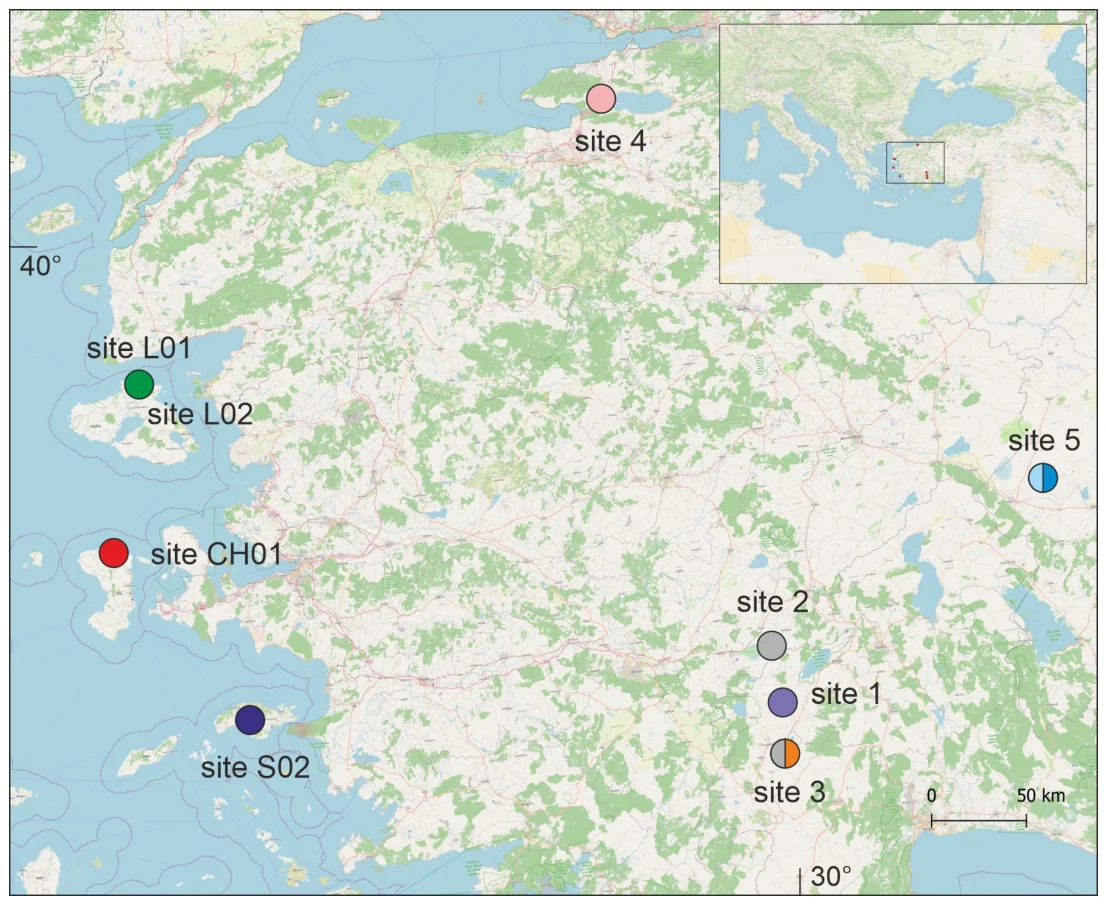
Map of the studied localities, showing the localities in West Anatolia (Türkiye) and the East Aegean islands. The geographical background is based on OpenStreetMap and shows major roads, watercourses, water bodies, and green areas. The inset shows the broader geographical position of the study area, with the area presented in the main map indicated by a rectangle. Geographical coordinates of sampling localities are given in the WGS84 reference system (EPSG:4326). Detailed locality information, including geographical coordinates and elevation, is provided in Table 1, while habitat descriptions are given in Section 2.1 (Materials and Methods). The colours of the locality symbols correspond to the mOTUs shown in Figure 3.

**Figure 2:**
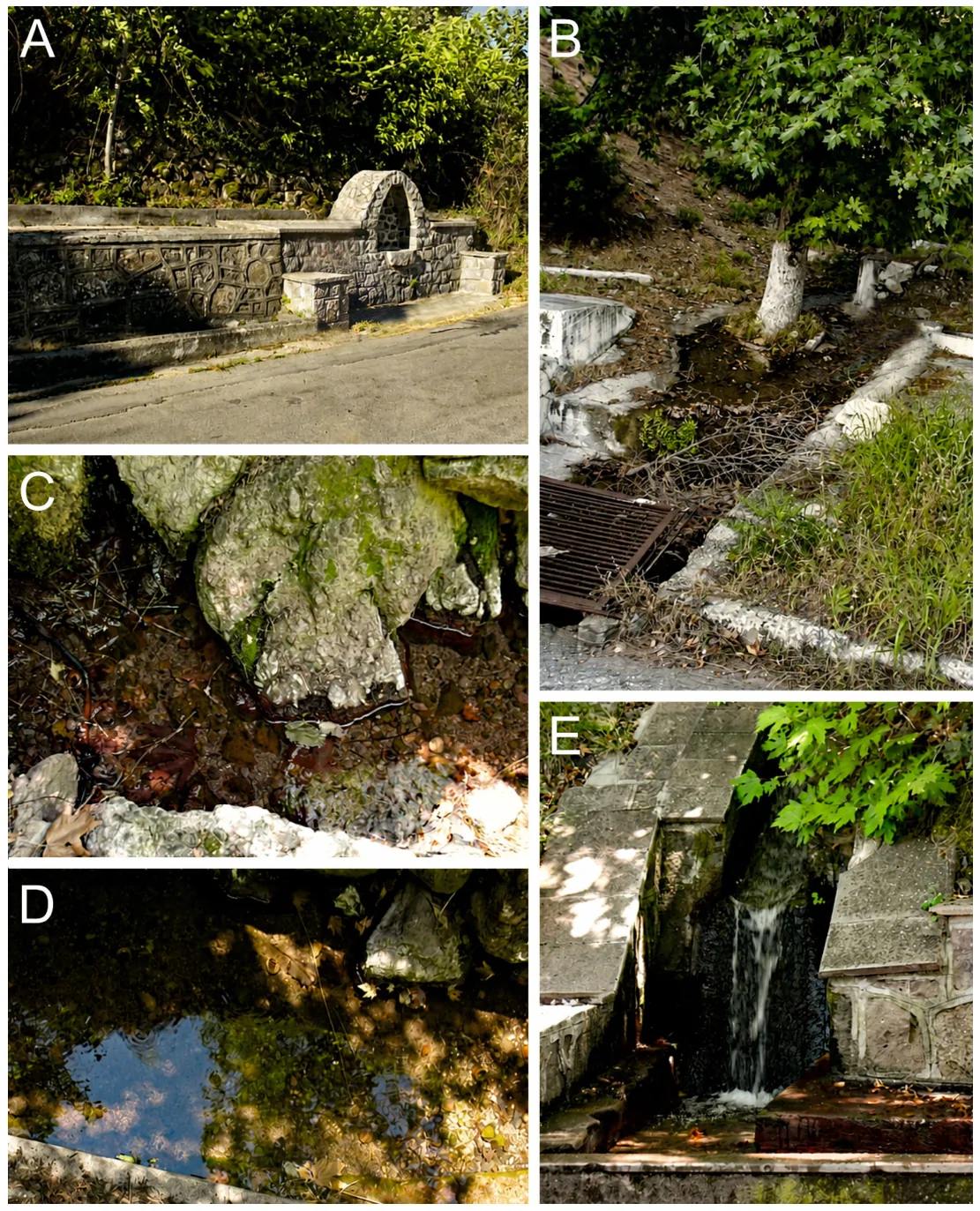
Localities: (**A**) type locality of *Pseudorientalia homeriana* sp. nov., Chios Island; (**B**) type locality of *Pseudorientalia pythagorasiana* sp. nov., Samos Island; (**C**,**D**) type locality of *Sapphonia lyrica* sp. nov., L01, Lesvos Island; (**E**) type locality of *S. lyrica*, L02, Lesvos Island.

The materials from the Aegean islands, already described in part [2,3], were collected in June of 2012, using a sieve (two meshes per mm), from four populations across three islands (Table 1, Figure 1 and Figure 2): CH01 Manna Nerou spring, near Ghiosonas, Chios Island (Figure 2A); L01, a spring near Klió, Epetimnos Mountain, Lesvos Island (Figure 2C,D); L02, a spring SSW (south–southwest) of Argennos Epetimnos Mountain, Lesvos Island (Figure 2E); and S02, Despoti Fountain between Koutsi and Pirgos, Samos Island (Figure 2B). The snails were newly collected, in May of 2026, by a 0.5 mm mesh net at five sampling locations, which are springs or spring-fed streams (Table 1; Figure 1 and Figure 2) in the south and southwest of Türkiye. Topotypes of *Pseudorientalia natolica* came from site 4, Pınarbaşı Spring, Gedelek Village of Bursa, Türkiye, 190 m a.s.l. Specimens of *Tefennia tefennica* and *Erosiconcha geldiayana* came from site 1. This site is the type locality of *Turcorientalia anatolica* Radoman, 1973 (not found there during our collection). *Graecoanatolica* cf. *tenuis* came from two localities (site 2 and 3).

The snails were washed twice in 80% ethanol and left to stand in this solution for around 12 h. Then the ethanol was changed twice more within 24 h and finally, after a few days, the 80% solution was replaced with a 96% one, in which the samples were stored at –20 °C.

### 2.2. Morphological Study

The shells were photographed with a CANON EOS 50D digital camera (Canon Inc., Tokyo, Japan). Adult males and females were dissected under a NIKON SMZ-U stereoscope microscope (Nikon Corporation, Tokyo, Japan), whose captured images were used to draw anatomical structures with a graphic tablet. Morphometric parameters of the shell were measured by one person using a Nikon DS-5 digital camera (Nikon Corporation, Tokyo, Japan) and the ImageJ 1.54g image analysis software [19]. Seven biometric parameters of the shell were measured, following the scheme of Falniowski et al. [20]. The penis and female genitalia (pallial oviduct) were examined under a light microscope (MOTIC) and photographed under a MOTIC microscope (Motic, Xiamen, China) with a dark field. The shells and opercula were cleaned with an ultrasonic cleaner. The radulae were extracted with Clorox™, applying the techniques described by [21], and examined and photographed using a HITACHI S-4700 scanning electron microscope (Hitachi High-Technologies Corporation, Tokyo, Japan).

Digital images were processed using tpsUtil v. 1.83 [22], and landmark coordinates were recorded in tpsDig2 v. 2.32 [23]. A total of 23 homologous landmarks were digitized on each shell. Geometric morphometric analyses were performed in MorphoJ v. 1.08.02 [24]. Landmark configurations were subjected to Generalized Procrustes Analysis to remove variation associated with specimen position, orientation, and scale. Principal Component Analysis (PCA) was subsequently performed on the covariance matrix of the Procrustes shape coordinates to summarize the major components of shell-shape variation and to visualize morphological similarities and differences among the analysed specimens. To enhance the descriptive PCA, differences in shell shape among taxa were quantified using Euclidean distances between mean Procrustes-aligned landmark configurations. An exploratory one-way Procrustes ANOVA was performed using 99,999 random permutations. Because sample sizes were small and unequal, heterogeneity of intra-group shape dispersion was additionally assessed by comparing bias-corrected distances of specimens to their respective group centroids using a permutation test (99,999 permutations). These additional analyses were performed on the Procrustes coordinates obtained from MorphoJ using a custom Python 3.15.2 script.

### 2.3. Molecular Laboratory Techniques

Whole DNA was extracted from ethanol-fixed tissues using the SHERLOCK^®^ kit (A&A Biotechnology, Gdańsk, Poland) according to the manufacturer’s procedure designated for fresh tissues. Extracted DNA was resuspended in 20 μL of Tris–EDTA (TE) buffer and stored for further analyses at −80 °C. Fragments of the mitochondrial cytochrome c oxidase subunit I (COI) gene and three nuclear genes, histone H3 (H3), 18S ribosomal RNA (18S), and 28S ribosomal RNA (28S), were amplified using the primers and PCR protocols described in [13]. Where possible, the nuclear markers were sequenced to test phylogenetic hypotheses based on COI and morphological data. PCR amplicons were purified using the Exo-SAP protocol and subsequently sequenced with the BigDye Terminator v3.1 Cycle Sequencing Kit (Applied Biosystems, Waltham, MA, USA). Sequencing products were purified with ExTerminator Columns (A&A Biotechnology, Gdańsk, Poland), after which DNA fragments were analysed on an ABI 3730xl DNA Analyzer (Genomed, Warsaw, Poland). The COI sequences of specimens collected from the Aegean islands in 2012 were obtained during previous studies and published by Szarowska et al. [2,3] in 2014. These previously obtained sequences were reused in the present phylogenetic analyses together with the new Turkish ones.

### 2.4. Molecular Data Analysis

Obtained COI sequences were preliminarily aligned using the MUSCLE algorithm [25] implemented in MEGA 7 [26], and the resulting alignments were manually verified in BIOEDIT 7.1.3.0 [27]. Uncorrected p-distances were calculated in MEGA 7 [26]. Substitution saturation was assessed with the test of Xia et al. [28] and Xia & Lemey [29], as implemented in DAMBE [30].

Phylogenetic analyses incorporated both newly generated and reference sequences obtained from previous studies (Table 2). In minute truncatelloid snails, DNA sequencing poses significant challenges, particularly when using old or poorly fixed material. Specimens stored at −20 °C become unsuitable for molecular studies after a few years. Furthermore, nuclear loci—which provide critical phylogenetic information—are even more difficult to sequence from older material due to their much lower copy numbers compared to mitochondrial DNA (mtDNA). Consequently, we were able to successfully sequence nuclear loci only from freshly collected specimens. The COI sequence of *Ecrobia maritima* (Milaschewitsch, 1916) (KX355835; [31]) was selected as an outgroup for the mitochondrial phylogenetic analysis, whereas the phylogenetic trees based on the nuclear markers (H3, 18S, and 28S) were left unrooted. Phylogenetic reconstruction was performed using Bayesian inference (BI) and maximum-likelihood (ML) approaches. The optimal nucleotide substitution models were determined in ModelTest-NG [32] through the raxmlGUI 2.0 interface [33]. Model selection was based on the corrected Akaike information criterion (AICc), which accounts for model complexity and includes a correction for finite sample size. Based on AICc, the TPM1uf+I+G4 model was selected for COI, TrN for H3, K80+I for 18S, and TIM2ef+I for 28S. Since these substitution models are not directly implemented in the classical RAxML framework, ML analyses were conducted under the more general GTR+Γ model for all four markers, with four discrete gamma rate categories to account for among-site rate heterogeneity. Maximum-likelihood analyses were carried out in RAxML-NG [34] using raxmlGUI 2, and bootstrap supports of the nodes were estimated with 1000 resampling. For COI Bayesian analyses were conducted in MrBayes v.3.2.3 [35] under default prior settings. Inference was conducted under the more general GTR+I+Γ model, with four discrete gamma rate categories. Two independent runs were performed simultaneously, each consisting of 10,000,000 generations and comprising one cold and three heated Markov chains. Analyses were initiated from random starting trees, with trees sampled every 1000 generations. The first 25% of sampled trees were discarded as burn-in, and the remaining trees were summarized in a 50% majority-rule consensus tree. Convergence diagnostics were assessed in Tracer v.1.7 [36]. Tree visualization and editing were performed in FigTree v.1.4.4 [37].

Species delimitation was evaluated using four approaches: Poisson Tree Processes (PTP and bPTP) [38], Automatic Barcode Gap Discovery (ABGD) [39], and Assemble Species by Automatic Partitioning (ASAP) [40]. All species delimitation analyses were conducted within the iTaxoTools 0.1 bioinformatics platform [41]. DeSignate [42] was used to distinguish species from another species, based on COI sequences, by determining the Molecular Diagnostic Characters (MDCs). Two types of characters were accepted: binary positions (the character state in the query group is different from the uniform character state in the reference group) and asymmetric positions (the character state in the query group is different from the non-uniform character state in the reference group). Positions of the MDCs refer to nucleotide positions in the COI alignment provided as Supplementary File S1.

**Table 2:** Reference sequences used for phylogenetic inference, with GenBank (GB) numbers and references.

Species	GB n/ref Numb/ref	18S	28S	H3	
*Adaniya cuneytsolaki* Odabaşı & Jaszczyńska, 2025	PX127888/[43]				
*Anadoludamnicola ayhani* Odabaşi, 2025	PX127890/[43]	OP099365/[9]			
*Ecrobia maritima* (Milaschewitsch, 1916)	KX355835/[31]	KY215947/[44]		MG551322/[45]	KY215953/[44]
*Erosiconcha geldiayana* (Schütt & Bilgin, 1970)	MZ606137/[4]	MZ605770/[9]	KY215953/[44]		
*Daphniola exigua* (A. Schmidt, 1856)	JF916470/[46]				
*Daphniola graeca* Radoman, 1973	EU047764/[47]				
*Daphniola louisi* Falniowski & Szarowska, 2000				MZ265364/[48]	
*Dicle bilgini* Falniowski & Odabaşi, 2025	PX127896/[43]				
*Graecoanatolica lacustristurca* Radoman, 1973	OP096234/[9]	OP099389/[9]			
*Graecoanatolica macedonica* Radoman & Stankovič, 1979	OP096235/[9]				
*Graecoanatolica pamphylica* (Schütt, 1964)	OP096236/[9]	OP099390/[9]			
*Graecoanatolica tenuis* Radoman, 1973	OP096237/[9]				
*Graecoanatolica vegorriticola* (Schütt, 1962)	KF758773/[49] KF758776/[49]	MZ605771/[4]			
*Grossuana angeltsekovi* Glöer & Georgiev, 2009	KU201090/[50]				
*Grossuana codreanui* (Grossu, 1946)	EF061920/[51]	EF061916/[51]			
*Iglicopsis butoti* Falniowski & Hofman, 2021	MW879273/[52]			MW865749/[52]	
*Iranobelgrandia persica* Falniowski, Grego & Fatemi, 2026	PZ391971/[53]				
*Kozanium torosum* Odabaşı & Falniowski, 2025	PV494927/[54]				
*Lerniana dianadelicadoae* Falniowski & Jaszczyńska, 2025	KC011734/[55]				
*Montenegrospeum bogici* (Pešić & Glöer, 2012)	KM875510/[56]	OK001219/[57]	OK001305/[57]	OK033490/[57]	OK001305/[53]
*Montenegrospeum sketi* Grego & Glöer, 2018	MG880216/[58]				
*Pseudorientalia tefennica* (Schütt & Yıldırım, 2003)	JX982802/[59]				
*Radomaniola curta curta* (Küster, 1853)	MZ539605/[60]	KC011724/[61]			
*Sardohoratia sulcata* Manganelli, Bodon, Cianfanelli, Talenti & Fo. Giusti, 1998	OP096316/[9]	OP099432/[9]			
*Sheitanok esmaae* Ekin & Odabaşi, 2025	PX127893/[43]	OP099434/[9]			
*Trichonia kephalovrissonia* Radoman, 1973	KC011728/[61]				

## 3. Results

### 3.1. Molecular Part

**Figure 3:**
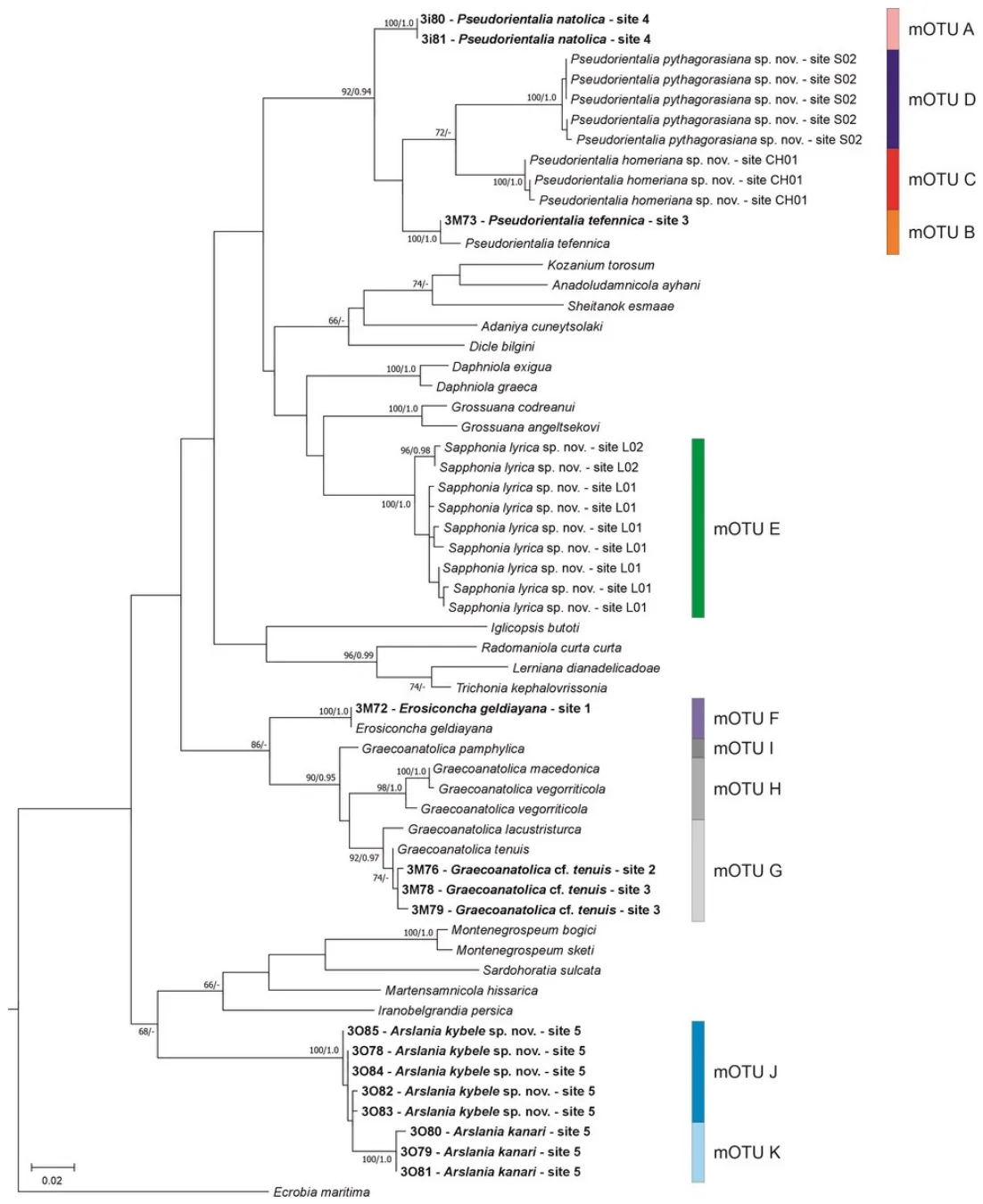
Maximum-likelihood tree based on the cytochrome c oxidase subunit I (COI) gene. Bootstrap supports (>70%), and Bayesian posterior probabilities (>0.95) are shown at the nodes. Coloured bars indicate the delimited mOTUs according to the four applied species delimitation methods. Newly obtained sequences are highlighted in bold.

We obtained 15 new sequences of COI (434 bp, GenBank accession numbers: QB223162–QB223176), H3 (164 bp, GenBank accession numbers: QB209838–QB209852), 18S (391 bp, GenBank accession numbers: QB212630–QB212644) and 28S (494 bp, GenBank accession numbers: QB212645–QB212659). For COI and H3, the test for the substitution saturation analysis has shown an ISS (0.182, 0.1519, respectively) that is significantly smaller than the critical ISS value (0.791, 0.6939, respectively), indicating that sequences were not saturated and thus useful in phylogenetic reconstruction. The topologies of the resulting phylograms were identical in both the ML and BI phylogram analyses; thus, we present the phylogram computed with RAxML. All species delimitation methods gave identical results (Figure 3). Our maximum-likelihood tree (Figure 3) clearly confirmed the monophyly of the well-supported subfamily Caspiinae B. Dybowski, 1913, but the other subfamily of the Hydrobiidae—Horatiinae D. W. Taylor, 1966—was not monophyletic in this tree. Deeper nodes, however, were not well supported; thus, the monophyletism of the Horatiinae remains open. A well-supported (bootstrap 92) clade clustered the type species of *Pseudorientalia* (*P. natolica*), as well as the snails from Chios and Samos Islands (sites CH01: mOTU C and S02: mOTU D), each of them as a distinct species (mOTUs C and D), but these Aegean taxa formed a well-supported (bootstrap 72) clade. Interestingly, *“Tefennia” tefennica* also belonged to the *Pseudorientalia* clade. The snails from the Lesvos Island (sites L01 and L02: mOTU E) did not belong to *Pseudorientalia*, but as mOTU E forms a clade representing a distinct genus, they formed a clade with *Grossuana* Radoman, 1983, and *Daphniola* Radoman, 1973, as sister taxa, although this relationship was not supported. The snails from the type locality of *Pseudorientalia kanari* were also clustered within the Horatiinae but not within the *Pseudorientalia* clade. They clustered within a nearly well-supported (bootstrap 68) clade with *Montenegrospeum* Pešić & Glöer, 2013 (type species *Bythiospeum bogici* Pešić & Glöer, 2012); *Sardohoratia* Manganelli, Bodon, Cianfanelli, Talenti & Fo. Giusti, 1998 (type species *Sardohoratia sulcata* Manganelli, Bodon, Cianfanelli, Talenti & Fo. Giusti, 1998); *Martensamnicola* Izzatullaev, Sitnikova & Starobogatov, 1985 (type species *Hydrobia (Amnicola) brevicula* E. von Martens, 1874); and *Iranobelgrandia* Falniowski, Grego et Fatemi, 2026 (type species *Iranobelgrandia persica* Falniowski, Grego et Fatemi, 2026). Within this clade another distinct species (mOTU J) was found. *Erosiconcha* and *Graecoanatolica* clustered within the Caspiinae. Close relationships between “*Tefennia*” and *Pseudorientalia*, as well as species distinctness of the two taxa assigned to the species *“Pseudrorientalia kanari*” so far, were confirmed by the phylogenetic inference applying the three nuclear loci: 18S, 28S, and H3 (see Supplementary Figure S2).

### 3.2. Shell Biometry in Pseudorientalia, Sapphonia gen. nov. and Arslania gen. nov.

**Figure 4:**
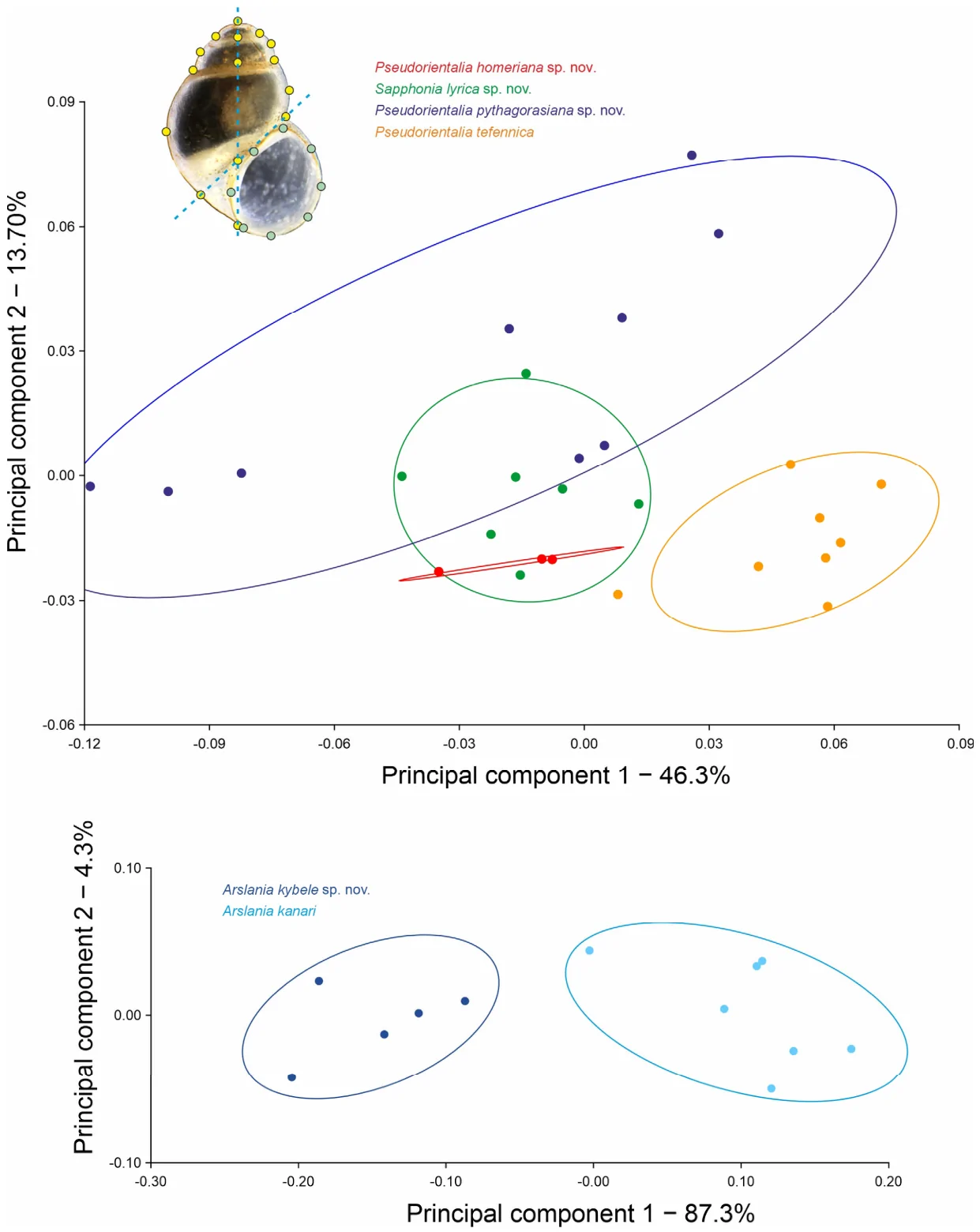
Principal component analysis (PCA) of shell shape in the analysed species of *Pseudorientalia*, *Sapphonia* gen. nov. and *Arslania* gen. nov. based on Procrustes-aligned landmark coordinates. Each point represents one specimen, and ellipses illustrate the distribution of specimens within taxa. The inset shows the positions of 23 landmarks used in the geometric morphometric analysis.

**Figure 5:**
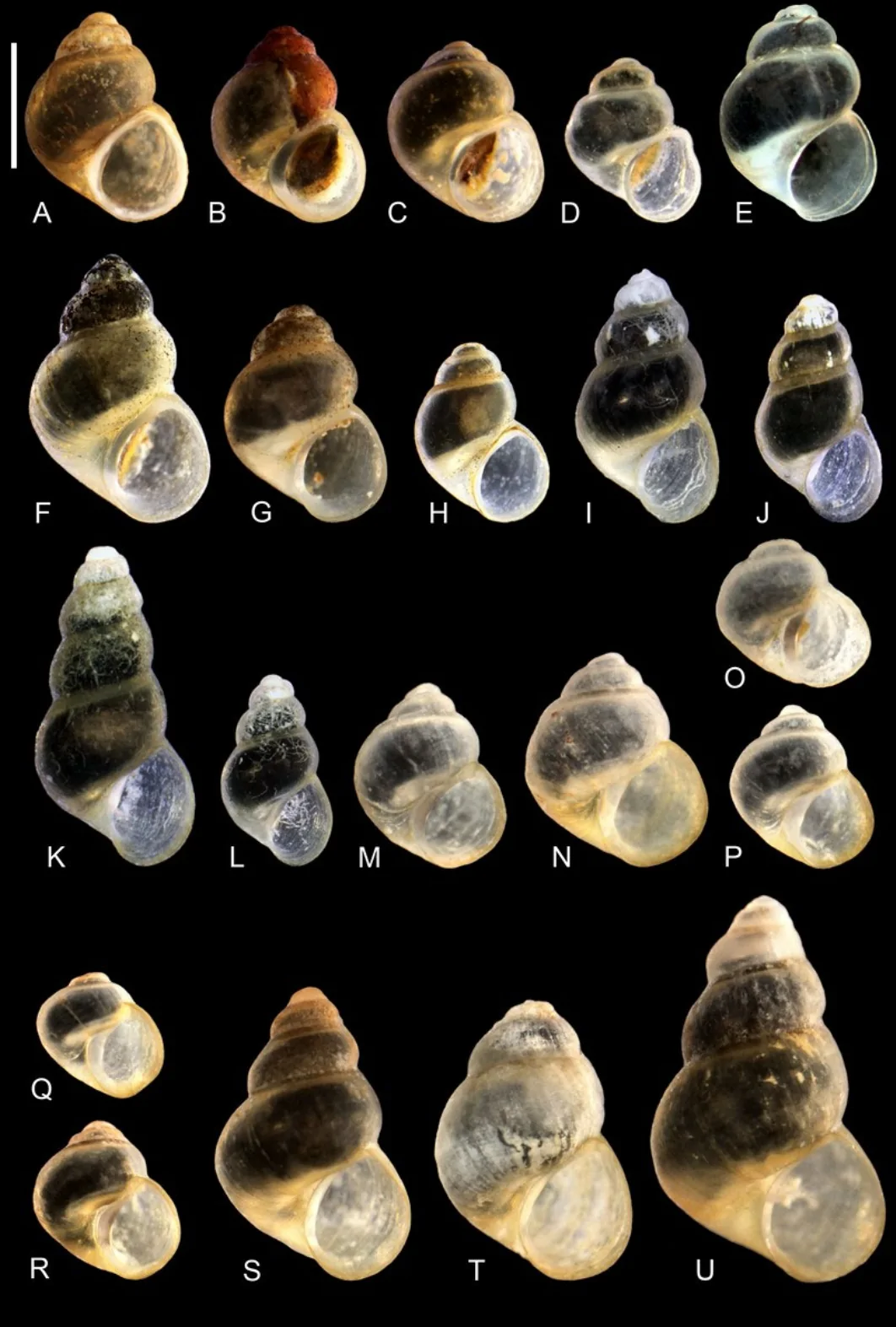
Shells of the studied snails: (**A**–**C**) *Pseudorientalia natolica*: (**A**) not sequenced, (**B**) 3i80, (**C**) 3i81; (**D**) *Pseudorientalia tefennica*, 3M73; (**E**) *Pseudorientalia homeriana* sp. nov., holotype; (**F**,**G**) *P. pythagorasiana* sp. nov.: (**F**) holotype, (**G**) another specimen with most frequent shell shape; (**H**) *Sapphonia lyrica* sp. nov., holotype; (**I**) *Erosiconcha geldiayana*, 3M72; (**J**–**L**) *Graecoanatolica* cf. *tenuis*: (**J**) 3M76, (**K**) 3M79, (**L**) 3M78; (**M**–**R**) *Arslania kybele* sp. nov.: (**M**) holotype, (**N**) 3O85, (**O**) 3O78, (**P**) 3O84, (**Q**) 3O82, (**R**) 3O83; (**S**–**U**) *Arslania kanari*: (**S**) 3O80, (**T**) 3O79, (**U**) 3O81. Bar equals 1 mm.

Principal component analysis revealed partial differentiation in shell shape among the analysed taxa (Figure 4 and Figure 5). The clearest morphological separation was observed between *P. homeriana* sp. nov. and *P. tefennica* comb. nova, while partial overlap occurred between *S. lyrica* sp. nov. and both *P. homeriana* sp. nov. and *P. pythagorasiana* sp. nov. *Pseudorientalia pythagorasiana* sp. nov. exhibited the greatest within-group shell-shape variation along both PC1 and PC2. In contrast, *P. homeriana* sp. nov. showed very limited variation, although this pattern may partly result from the small number of specimens analysed (*n* = 3). *Pseudorientalia tefennica* showed moderate variation, whereas *Sapphonia lyrica* sp. nov. exhibited relatively restricted variation in shell shape, overlapping *Pseudorientalia homeriana* sp. nov. and *P. pythagorasiana* sp. nov., which confirms observable lack of conchological differences between *Sapphonia* gen. nov. and *Pseudorientalia*. Pairwise distances between mean Procrustes-aligned landmark configurations ranged from 0.0457 to 0.0891. The smallest distance was observed between *P. homeriana* sp. nov. and *S. lyrica* sp. nov. (0.0457), despite their assignment to phylogenetically distinct genera. These results indicate that considerable similarity in shell shape may occur between phylogenetically distinct lineages, supporting the limited taxonomic value of shell morphology in these hydrobiid snails. However, these patterns should be interpreted cautiously given the relatively small sample sizes.

In the second dataset, the PCA indicated a clear differentiation in shell shape between *Arslania kybele* sp. nov. and *A. kanari*, primarily along PC1, whereas their ranges of PC2 scores largely overlapped the Euclidean distance between their mean Procrustes-aligned landmark configurations, which was 0.2536, indicating considerable differentiation in mean shell shape. However, these results should be interpreted cautiously given the relatively small sample sizes.

### 3.3. Systematic Parts

**Truncatelloidea Gray, 1840**

**Hydrobiidae W. Stimpson, 1865**

**Horatiinae D. W. Taylor, 1966**

**Genus *Pseudorientalia* Radoman, 1983**

***Paludina natolica* Küster, 1853 (type)**

GenBank sequence numbers: QB223162–QB223163 Locality 4 (Table 1; Figure 1)

**Shell**: (Figure 5A–C) up to 2.18 mm high, ovate-conical, broad, with spire relatively low, with about 4 whorls, the spire’s height about 19% of the shell height, body whorl bulky and massive. Teleoconch whorls moderately convex, evenly rounded, growing regularly in diameter. Aperture rather broad, ovoid, outer lip simple, parietal lip complete and broad, umbilicus slit-like or absent. Teleoconch moderately thick-walled, its wall translucent, with delicate growth lines, periostracum yellowish or brownish. Operculum smooth on both surfaces.

**Figure 6:**
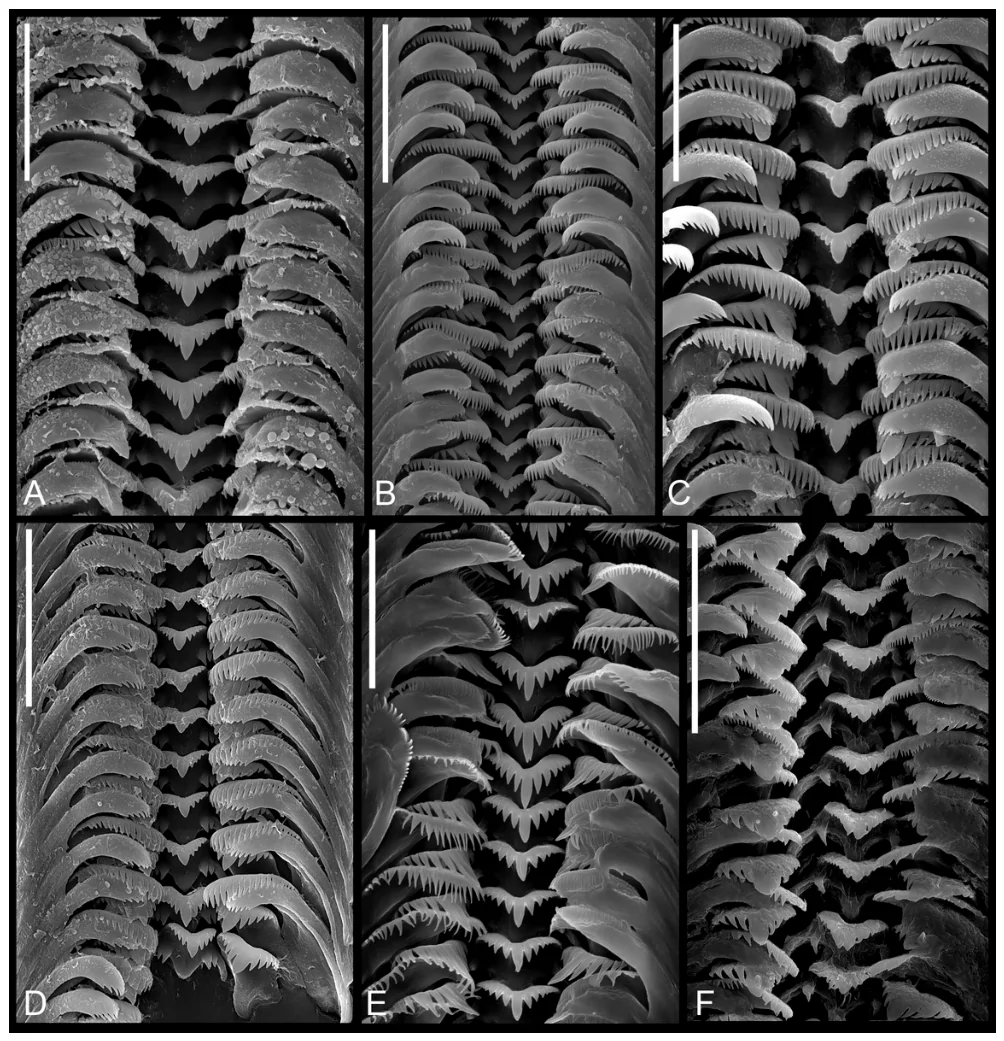
Radulae of the studied species: (**A**) *Pseudorientalia natolica*, (**B**) *Sapphonia lyrica* sp. nov., (**C**) *Erosiconcha geldiayana*, (**D**) *Graecoanatolica* cf. *tenuis.* (**E**) *Arslania kybele* sp. nov., (**F**) *Arslania kanari*. Bar equals (**A**–**C**) 20 µm, (**D**) 30 µm, (**E**) 20 μm, (**F**) 40 μm.

**Radula** (Figure 6A): central tooth with lateral angles highly expanded, ventral process well excavated, median cusp blunt and massive, adjacent cusps on the cutting edge short (less than half of the central cusp length), blunt, most lateral of them blunt and slightly separated one from the other [this may explain the opinion of Radoman [62] about their unusually small size], basal cusps moderately big, located relatively low on lateral angles of central tooth. Central tooth formula:

$$\frac{5-1-5}{1-1}$$

Lateral tooth following formula: 3 − 1 − 6, cusps much bigger than on the central tooth, broad and massive, the biggest one twice longer and more than twice broader than the adjacent ones. Inner marginal tooth with 22 − 27 delicate and slender cusps, outer marginal tooth with about 15 delicate sharp cusps.

**Soft parts morphology and anatomy**: Head and mantle intensely pigmented black, ctenidium present.

**Figure 7:**
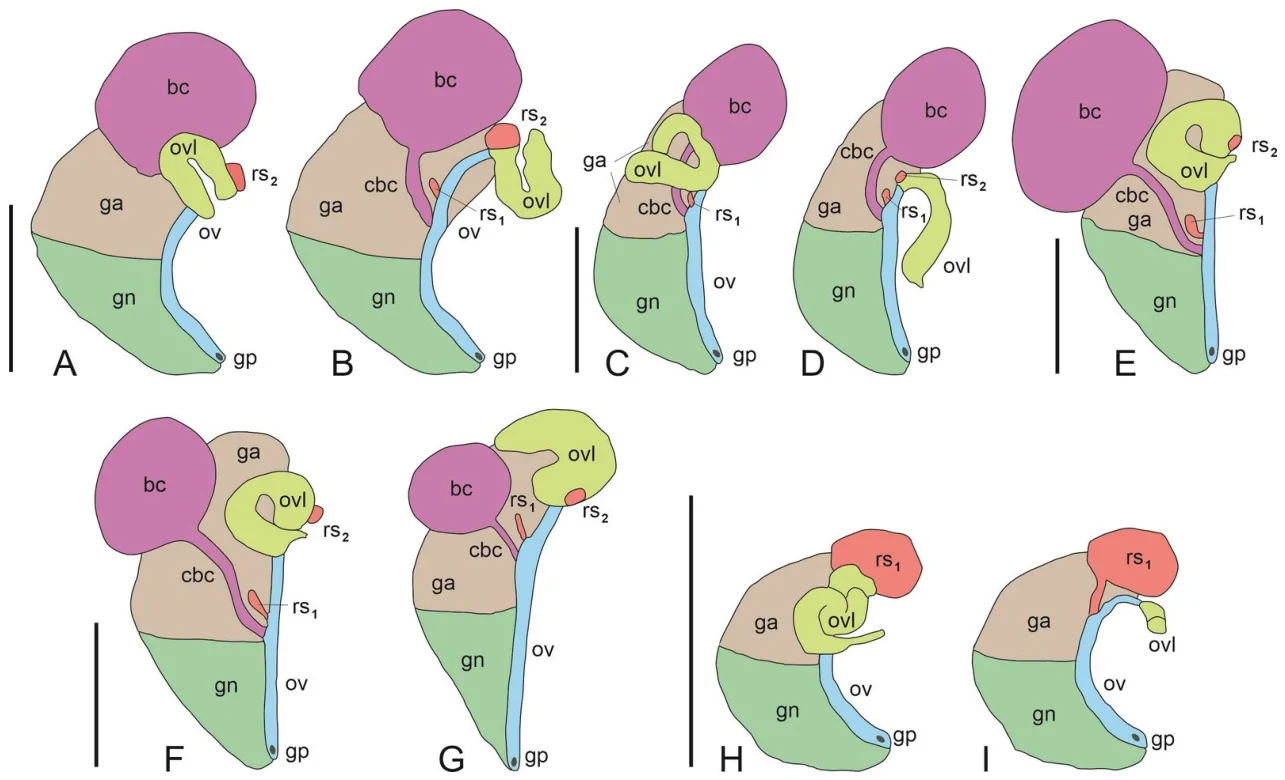
Female reproductive organs: (**A**,**B**) *Pseudorientalia natolica*, (**C**,**D**) *Pseudorientalia homeriana* sp. nov., (**E**) *Pseudorientalia pythagorasiana* sp. nov., (**F**,**G**) *Sapphonia lyrica* sp. nov., (**H**,**I**) *Graecoanatolica* cf. *tenuis*. Bars at left side of figures equals: 0.5 mm. Abbreviations: bc—bursa copulatrix, cbc—duct of bursa copulatrix, ga—albuminoid gland, gn—nidamental gland, gp—gonoporus, ov—oviduct, ovl—loop of (renal) oviduct, rs_1_—distal receptaculum seminis, rs_2_—proximal receptaculum seminis.

**Female reproductive organs** (Figure 7A,B) as described and figured by Radoman [62], with big and bulky bursa copulatrix and two receptacula seminis, the distal one small, nearly rudimentary.

**Figure 8:**
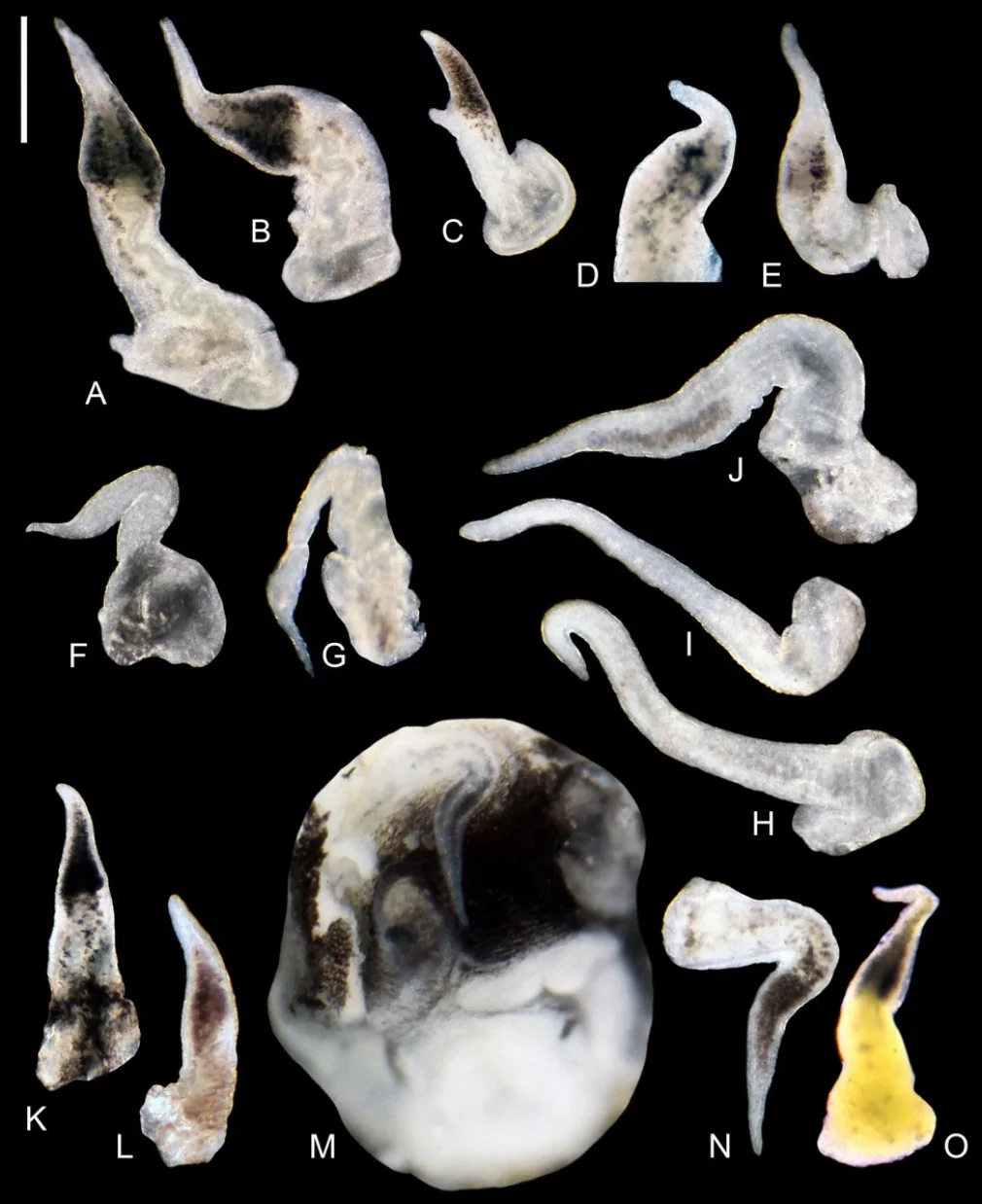
Penes of the studied snails: (**A**,**B**) *Pseudorientalia natolica*; (**C**) *Pseudorientalia tefennica*; (**D**) *Pseudorientalia homeriana* sp. nov.; (**E**) *Pseudorientalia pythagorasiana* sp. nov.; (**F**,**G**) *Sapphonia lyrica* sp. nov.; (**H**,**I**) *Erosiconcha geldiayana*; (**J**) *Graecoanatolica* cf. *tenuis*; (**K**,**L**) *Arslania kybele* sp. nov.; (**M**–**O**) *Arslania kanari*. Bar equals 0.2 mm.

**Penis** (Figure 8A,B) gradually tapering, its base broad and with small outgrowth proximally on the left side, in its distal part intensely pigmented black, vas deferens visible inside the penis.

**Remarks**: Our locality in Gedelek is about 12 km far from the type of locality of Küster [63], and all the character states confirm our identification of this type species of *Pseudorientalia*.

***Pseudorientalia tefennica* (Schütt et Yıldırım, 2003)**

*Tefennia tefennica* Schütt et Yıldırım, 2003

*Tefennia tefennica* in Çağlan et al. (2012) [59]

GenBank sequence numbers: QB223164

Locality 1 (Table 1; Figure 1)

The shell of *T. tefennica* (Figure 4 and Figure 5D), with characteristically sinuated outer lip, corresponds to the description of the species and drawings by Schütt & Yildirin [64] and Çağlan et al. [59]. The radula as presented in [59]. Penis (Figure 8C) gradually tapering with small (but bigger than in *P. anatolica*) outgrowth on the left side of its median part, similar to the ones described and illustrated in [59,64].

***Pseudorientalia homeriana* Hofman, sp. nov.**

*Pseudorientalia* sp. from the locality CH01 in Szarowska et al. [3]; Figure 1

LSID urn:lsid:zoobank.org:act:31AC2BF3-3B8C-463F-ACF0-7619416AA4B0

GenBank sequence numbers: KJ920492, KJ920493, and KJ920497

Locality CH01 (Table 1; Figure 1)

**Holotype**: Ethanol-fixed specimen (Figure 5E) collected on the 23th of June 2012 in a spring Manna Nerou Spring, near Ghiosonas, Chios Island (Figure 1): 38°33′28.7″ N, 26°04′50.2″ E, 30 m a.s.l., by Magdalena Szarowska and Andrzej Falniowski. Zoological Museum of Jagiellonian University, Kraków, Poland, voucher number ZMUJ/2026/14.

**Paratypes**: 15 ethanol-fixed specimens, collected at the type locality (a spring Manna Nerou Spring, near Ghiosonas, Chios Island (Figure 2A): 38°33′28.7″ N, 26°04′50.2″ E, 30 m asl), voucher numbers ZMUJ/P/2026/15-29.

**Description**: Shell (Figure 5E and Supplementary Figure S1A–C) up to 1.53 mm high, ovate-conical, very broad, with spire low, with about 5 whorls, spire height 19–25% of the shell height. Teleoconch whorls slightly convex, evenly rounded, growing regularly in diameter. Aperture rather narrow, ovoid, outer lip simple, parietal lip complete and narrow, umbilicus slit-like or moderately broad. Teleoconch moderately thick-walled, its wall translucent, with delicate growth lines, periostracum yellowish or whitish. Shell measurements: Figure 4. Operculum paucispiral, smooth on both surfaces.

**Radula**: unknown

*Soft parts morphology and anatomy*: Head and mantle pigmented intensely black, ctenidium present.

**Female reproductive organs**: (Figure 7C,D) with long and narrow pallial accessory gland complex (albuminoid and nidamental gland), big and bulky ellipsoid bursa copulatrix expanding in size over the gland complex and with well discernible duct, moderately broad U-shaped loop of oviduct, and two receptacula seminis, proximal and distal, both small in size.

**Penis** (Figure 8D) rather moderately broadly triangular than gradually tapering, without any outgrowths, distally narrow, black pigment at the dorsal surface at its median part, vas deferens partly visible inside.

**Differential diagnosis**: *Pseudorientalia certi* differs from *P. homeriana* sp. nov. in the shell with wider parietal lip and umbilicus, and penis less triangular in shape, with terminal spot of lack pigment [65]. *P. intranodosa* differs in less oval and less broad shell outline, shell with narrower aperture, and penis with a big spot of black pigment at its terminal part [66]. The holotype of *P. homeriana* sp. nov. resembles the one of *P. kanari*, but most of the shell of *P. homeriana* sp. nov. is more oval in shape. In *P. kanari* penis is long and narrow, with a spot of black pigment terminally, accessory gland complex of the female reproductive organs longer, albuminoid gland less broad, proximal receptaculum seminis bigger, loop of oviduct less thick [67] than in *P. homeriana* sp. nov. *P. natolica* differs from *P. homeriana* sp. nov. in less oval shell outline, penis gradually tapering not moderately broadly triangular like in *P. homeriana* sp. nov., female reproductive organs in *P. natolica* have bigger bursa copulatrix and proximal receptaculum seminis. *P. pythagorasiana* sp. nov. differs from *P. homeriana* sp. nov. in more oval shell outline, gradually tapering penis, and female reproductive organs with shorter accessory glands complex, much bigger bursa copulatrix and distal receptaculum seminis, and more massive loop of oviduct. The MDCs in comparison with all *Pseudorientalia* sequences used in phylogenetic analysis: binary: 92(G), 93(C), 101(C), 102(T), 146(G), 147(C), 209(G), 212(G), 377(G), asymmetric: 86(A), 359(G).

**Derivation of name**: The species name honours the great poet Homer, connected to Chios Island through a tradition that dates back to ancient times. According to tradition, Daskalopetra (the stone of the teacher in Greek), known also as Homerus Stone, a historical place in the area of Vrontados on Chios, according to the long-lasting tradition, is actually the stone where Homer was seated on when narrating his poems, since, being probably blind, he was not able to write them.

**Distribution and habitat**: The species is known only from the type locality so far.

***Pseudorientalia pythagorasiana* Jaszczyńska, sp. nov.**

*Pseudorientalia* sp. from the locality S02 [3]: Figure 1

LSID urn:lsid:zoobank.org:act:17C7AEB0-6534-40AC-8E9C-69232CB58A0D

GenBank sequence numbers: KJ920473- KJ920477

Locality S02 (Table 1; Figure 1)

**Holotype:** Ethanol-fixed specimen (Figure 5F) collected on the 20th of June 2012 at Despoti Fountain (Despoti Pigadi) between Koutsi and Pirgos, Samos Island (Figure 2B, locality S02): 37°42′59″ N, 26°46′51″ E, 490 m a.s.l., by Magdalena Szarowska and Andrzej Falniowski. Zoological Museum of Jagiellonian University, Kraków, Poland, voucher number ZMUJ/2026/30.

**Paratypes**: 15 ethanol-fixed specimens, collected at the type locality (Despoti Fountain between Koutsi and Pirgos, Samos Island: 37°42′59″ N, 26°46′51″ E, 490 m a.s.l.) voucher numbers ZMUJ/P/2026/31-45.

**Description**: Shell (Figure 5F,G and Supplementary Figure S1D–G) up to 2.28 mm high, ovate-conical, moderately broad or broad, with spire moderately low or low, with about 5 whorls, spire height 13–27% of the shell height. Teleoconch whorls slightly convex, evenly rounded, growing regularly in diameter. Aperture rather narrow or broader, ovoid, outer lip simple, parietal lip complete and narrow or broader, umbilicus slit-like. Teleoconch moderately thick-walled, its wall translucent, with delicate growth lines, periostracum yellowish or whitish. Shell measurements: Figure 4. Operculum paucispiral, smooth on both surfaces.

**Radula** [2]: Figures 14–16: with many cusps on each tooth, central tooth with lateral angles highly expanded, ventral process well excavated, median cusp sharp and slender, adjacent cusps on the cutting edge short (less than half of the central cusp length), sharp, well separated from each other, basal cusps moderately long and slender, the other cusps on the rhachis rather sharp and tiny. Central tooth formula:

$$\frac{5(6)–1–5(6)}{1\text{ – }1}$$

(sixth cusp rudimentary, if present). Lateral tooth with various numbers of cusps, its formula: 3 − 1 − 5, or 5 − 1 − 6, or 2 − 1 − 4, cusps sharp and longer than on the central tooth, biggest cusp much less than twice as long as adjacent ones. Inner marginal tooth with 22 − 29 delicate and slender cusps, outer marginal tooth with about ten small cusps.

**Soft parts morphology and anatomy**: Head and mantle pigmented intensely black, ctenidium present.

**Female reproductive organs**: (Figure 7E) with short and broad pallial accessory gland complex (albuminoid and nidamental gland), extremely big and bulky ellipsoid bursa copulatrix with well discernible long duct, nearly all bursa situated outside the gland complex, broad U-shaped loop of oviduct, and two receptacula seminis, proximal and distal, proximal small, distal bigger.

**Penis** (Figure 8E) rather moderately broadly triangular than gradually tapering, without any outgrowths, distally narrow, spot of black pigment at the dorsal surface at its median part, vas deferens partly visible inside.

**Differential diagnosis**: *Pseudorientalia certi* differs from *P. pythagorasiana* sp. nov. in the shell with wider parietal lip and umbilicus, and penis with terminal spot of lack pigment and no pigments on its median part [65]. *P. intranodosa* differs in shell—shell oval, less broad, with aperture narrower than in *P. pythagorasiana* sp. nov., and penis with big spot of black pigment at terminal part, no pigment in the median part [66]. *P. kanari* differs in the more conic shell in most of specimens (although not in holotype), penis slenderer, with a spot of black pigment terminally, not medially as in *P. pythagorasiana* sp. nov., female reproductive organs with accessory gland complex longer, albuminoid gland less broad, bursa copulatrix smaller, proximal receptaculum seminis bigger, loop of oviduct less thick [67]. *P. natolica* differs in the shell less oval in outline, penis with outgrowth on its left side, with a spot of black pigment terminally, not medially as in *P. pythagorasiana* sp. nov., female reproductive organs with bursa copulatrix smaller, proximal receptaculum seminis bigger, and distal one smaller than in *P. pythagorasiana* sp. nov. The MDCs in comparison with all *Pseudorientalia* sequences used in phylogenetic analysis: binary: 59(C), 65(G), 95(G), 98(G), 134(G), 162(C), 170(G), 173(G), 203(G), 221(C), 227(C), 245(C), 266(C), 344(C), 414(C); asymmetric: 62(G), 86(C), 224(A), 359(A).

**Derivation of name**: The species name honours Pythagoras the great ancient Greek mathematician and philosopher born and living in Samos. There is the Cave of Pythagoras where Pythagoras was hiding when the tyrant of Samos, Polycrates, was chasing after him. The cave is located on the eastern side of Mount Kerkis, the highest mountain of the Aegean.

**Distribution and habitat**: The species is known only from the type locality so far.

**Genus *Sapphonia* Szarowska et Falniowski gen. nov.**

LSID urn:lsid:zoobank.org:act:E1CEDC19-0284-4906-982E-954CD1EA3DAD

Type species: *Sapphonia lyrica* sp. nov. by monotypy.

**Diagnosis**: a minute representative of Horatiinae, with very broad ovate conical shell with low spire, central tooth of radula with one pair of basal cusps, on lateral tooth cusps massive, big bulky bursa copulatrix, two receptacula seminis distal one bigger, penis gradually tapering with small flat outgrowth on the left of its proximal part, distally forming a filament.

**Derivation of name**: Genus’s name to honour Sappho, a great ancient Greek poet, representing aeolian lyric, born and writing on Lesbos Island. Her lyrics are still read.

**Differential diagnosis**: The MDCs in comparison with all analysed sequences: binary: 353(G).

***Sapphonia* *lyrica* Szarowska et Falniowski sp. nov.**

*Pseudorientalia* sp. from the localities L01 and L02 in [3]: Figure 1

LSID urn:lsid:zoobank.org:act:A40318A9-07F1-4CDF-B6AC-BA5F86FD419A

GenBank sequence numbers: KJ920478- KJ920489

Locality L01 and L02 (Table 1, Figure 1)

**Holotype**: Ethanol-fixed specimen (Figure 5H) collected on the 25 June 2012 at a spring near Klió, Epetimnos Mountain, Lesvos Island (Figure 2C,D; locality L01 in [3]: 39°21′30.5″ N, 26°18′39.7″ E, 208 m a.s.l., by Magdalena Szarowska and Andrzej Falniowski. Zoological Museum of Jagiellonian University, Kraków, Poland, voucher number ZMUJ/2026/46.

**Paratypes**: 20 ethanol-fixed specimens, collected at the type locality (spring near Klió, Epetimnos Mountain, Lesvos Island: 39°21′30.5″ N, 26°18′39.7″ E, 208 m a.s.l.), voucher numbers ZMUJ/P/2026/47-66.

**Description**: Shell (Figure 5H and Supplementary Figure S1H–N) up to 1.91 mm high, ovate-conical, very broad, with spire low, with about 4 whorls, spire height 17–22% of the shell height. Teleoconch whorls slightly convex, evenly rounded, growing regularly in diameter. Aperture rather narrow, ovoid, outer lip simple, parietal lip complete and narrow to moderately broad, umbilicus slit-like or absent. Teleoconch moderately thick-walled, its wall translucent, with delicate growth lines, periostracum whitish. Shell measurements: Figure 4. Operculum paucispiral, smooth on both surfaces.

**Radula** (Figure 6B): central tooth with lateral angles highly expanded, ventral process well excavated, median cusp blunt and slender, adjacent cusps on the cutting edge short (less than half of the central cusp length), blunt, most lateral of them slightly separated one from the other, basal cusps small. Central tooth formula:

$$\frac{5\left(6\right)-1-5\left(6\right)}{1-1}$$

Lateral tooth following formula: 5 − 1 − 6, cusps much bigger than on the central tooth, slender, the biggest one twice longer and more than twice broader than the adjacent ones, and than the median cusp on the central tooth. Inner marginal tooth with about 27 long. Delicate and slender cusps, outer marginal tooth with about 12 delicate sharp cusps.

**Soft parts morphology and anatomy**: Head and mantle pigmented intensely black, ctenidium present.

**Female reproductive organs**: (Figure 7F,G) with long and narrow pallial accessory gland complex (albuminoid and nidamental gland), big and bulky nearly spherical bursa copulatrix with well discernible long duct, expanding outside the gland complex, broad U-shaped loop of oviduct, and two receptacula seminis, proximal and distal, proximal small, distal bigger.

**Penis** (Figure 8F,G) gradually tapering but sometimes broad at its proximal part, with small flat outgrowth on the left of its proximal part, distally narrow, forming a filament, some black pigment on its proximal part in some specimens, vas deferens partly visible inside.

**Differential diagnosis**: In the well supported clade, *Grossuana* Radoman, 1983 and *Daphniola* Radoman, 1973 are grouped with *Sapphonia lyrica* sp. nov. (Figure 3). *Daphniola* differs from *Sapphonia* gen. nov. by its valvatoid shell, dwarf distal receptaculum seminis and penis with much bigger outgrowth on its left side [68]. *Grossuana* has similar shell, but its bursa copulatrix is much smaller, proximal receptaculum bigger, and distally there is rather big, bi-lobed outgrowth on the left side [51], not present in *Sapphonia* gen. nov. The MDCs in comparison with all analysed sequences: binary: 341(C), asymmetric: 284(G), 317(C).

**Derivation of name**: The species is an epithet that honours Sappho’s lyrics, still a classic one.

**Distribution and habitat**: Apart from the type locality, this species was also found in a spring SSW of Argennos, Epetimnos Mountain, Lesvos Island (Site L02; Figure 1 and Figure 2E), 39°21′41.1″ N, 26°16′14.7″ E, 362 m a.s.l., situated not far from the type locality.

**Genus *Arslania* D. A. Odabaşı, gen. nov.**

LSID urn:lsid:zoobank.org:act:CEF4ECDB-57AA-4A5A-BE1C-834871A7234E

Type species *Pseudorientalia kanari* D. A. Odabaşı, 2024

*Arslania kanari* (D. A. Odabaşı, 2024)

**Diagnosis**: a minute representative of Horatiinae with conical, ovate-conical or trochoid semi-transparent shell with slowly growing slightly convex whorls, one pair of big basal cusps of the central tooth of the radula, medium-sized bulky bursa copulatrix, two receptacula seminis, distal minute, proximal thick thread-shaped, penis gradually tapering with no outgrowths, intensely black pigmented from its middle to the tip, vas deferens visible inside unpigmented proximal part of the penis.

**Differential diagnosis**: as stated above, *Arslania*’ sister clade consisted of *Martensamnicola*, endemic to Central Asia [69], *Sardohoratia*, endemic to Sardinia [70], and *Montenegrospeum*, endemic to the former Yugoslavia [20]. All these four genera share two receptacula seminis in typical positions of rs_1_ and rs_2_ as defined by Radoman [62,71], and bursa copulatrix expanded over the proximal part of the albumen gland [53,56,70,72]. *Montenegrospeum* and *Iranobelgrandia* differ from all the other three genera in turriform shell and a distinct lobe of the left side of the penis [53,56,73], lobeless in the other three genera. In *Montenegrospeum* penis is gradually tapering and slim, its filament narrow, in *Iranobelgrandia* broadly triangular, with broad and not prominent outgrowth on its left side. *Sardohoratia* differs from all the other three genera in its valvatoid shell, no eye spots and pigment. Its bursa copulatrix is similarly large as in *Arslania* gen. nov., receptaculum equal or slightly bigger than the proximal one [70]. In *Martensamnicola* and *Arslania* gen. nov. the shell is conical or ovate-conical, In *Martensamnicola* bursa copulatrix is much smaller than in *Arslania* gen. nov., two moderately big receptacula similar in size (in *Arslania* gen. nov. distal one minute), penis broadly triangular, not gradually tapering [72]. In *Iranobelgrandia*, similarly as in *Arslania*, proximal receptaculum seminis is big, distal one dwarf [53]. Basal cusps on the central tooth in *Arslania* gen. nov. are much bigger than in *Martensamnicola* [69] and *Iranobelgrandia* [53], similarly big but sharper than in *Sardohoratia* [70]. The MDCs in comparison with all analyzed sequences: binary: 341(C), asymmetric: 284(G), 317(C).

**Derivation of name**: The generic name *Arslania* honours Dr. Naime Arslan, who first discovered and collected the population representing this genus, in recognition of her valuable contributions to the study of Turkish freshwater molluscs.

**Distribution and habitat**: The species is known from only its type locality: Göksu Spring, near Göktepe village, Sivrihisar district, southeast of Eskişehir province, Türkiye.

***Arslania kanari* (D. A. Odabaşı, 2024)**

*Pseudorientalia kanari* D. A. Odabaşı, 2024

GenBank sequence numbers: QB223170-QB223172

Locality 5 (Table 1; Figure 1)

**Shell**: (Figure 5S,U) as described [67], up to 3.25 mm high, conical or ovate-conical, moderately broad, with spire high or moderately low, with about 4½ whorls, the spire’s height 32–38 (but only 24 in some specimens: Figure 5T) of the shell height, body whorl moderately massive, teleoconch whorls moderately convex, evenly rounded, growing regularly in diameter. Aperture rather narrow, ovoid, with an angle on its right upper corner, outer lip simple, parietal lip complete and moderately broad, umbilicus slit-like or absent. Teleoconch moderately thick-walled, its wall translucent, with delicate growth lines, periostracum pale yellowish or brownish. Shell measurements: Figure 4. Operculum paucispiral. smooth on both surfaces.

**Radula** (Figure 6F): central tooth with lateral angles very highly expanded, ventral process well excavated, median cusp blunt and short, slightly longer but much broader than the adjacent cusps on the cutting edge, which are short and blunt, most lateral of them blunt and slightly separated one from the other, basal cusps very big and sharp, located relatively high on lateral angles of central tooth. Central tooth formula:

$$\frac{8-1-8}{1-1}$$

Lateral tooth following formula: 4 − 1 − 4, cusps much bigger than on the central tooth, broad and massive, the biggest one broad and blunt, twice longer and much more than twice broader than the adjacent ones. Inner marginal tooth with about 32 delicate and slender cusps, outer marginal tooth with about 15 delicate sharp cusps.

**Soft parts morphology and anatomy**: Head and mantle intensely pigmented black, ctenidium present.

**Female reproductive organs**: as described and figured by Odabaşı et al. [67], with big and bulky bursa copulatrix and two receptacula seminis, the distal one small, nearly rudimentary, the proximal one big, thread-like.

**Penis** (Figure 8M–O) gradually tapering with no outgrowths, moderately thick at its base, intensely black pigmented from its middle to the tip, vas deferens visible inside unpigmented proximal part of the penis.

***Arslania kybele* D. A. Odabaşı, sp. nov.**

LSID urn:lsid:zoobank.org:act:F744090A-B2E2-4A7A-88BD-C94DAFB6003E

GenBank sequence numbers: QB223169, QB223173-QB223176

Locality 5 (Table 1, Figure 1)

**Holotype**: Ethanol-fixed specimen (Figure 5M), collected on 08-05-2026 at Göksu Spring, Göktepe village, Sivrihisar district, southeast of Eskişehir province, Türkiye (Table 1, Figure 1): 39°9′1.4″ N, 31°36′49.32″ E, by D. A. Odabaşı, deposited at the Limnology Museum of Çanakkale Onsekiz Mart University (COMULM) under the voucher code of COMULM-G 300.

**Paratypes**: A total of 165 ethanol-fixed specimens were collected from the type locality (Göksu Spring, Göktepe Village, Sivrihisar District, southeastern Eskişehir Province, Türkiye; 39°09′01.4″ N, 31°36′49.32″ E). Of these, 90 specimens are deposited in COMULM (COMULM-G 301), and 75 specimens are deposited in the Hydrobiology Museum, Eskişehir Osmangazi University (ESOGU), Türkiye (ESOGU Gastropoda (C) No. 3).

**Description**: Shell (Figure 5M–R) up to 1.76 mm high, trochoid, very broad, with spire low, with about 4 whorls, spire height 10–18% of the shell height. Teleoconch whorls slightly convex, evenly rounded, growing rather abruptly in diameter. Aperture rather broad, ovoid, with an angle on its right upper corner, outer lip simple, parietal lip complete and broad, covering umbilicus, teleoconch strikingly thick-walled, its wall translucent, with growth lines delicate, periostracum whitish. Shell measurements: Figure 4. Operculum paucispiral, smooth on both surfaces.

**Radula** (Figure 6E): central tooth with lateral angles very highly expanded, ventral process well excavated, median cusp blunt and narrow, twice longer and slightly broader than the adjacent cusps on the cutting edge, which are elongate triangular in shape and sharp, well separated one from the other, basal cusps big, located relatively high on lateral angles of central tooth. Central tooth formula:

$$\frac{\left(8\right)7-1-7\left(8\right)}{1-1}$$

Lateral tooth following formula: 5 − 1 − 8, cusps somewhat longer than the ones on the central tooth, narrow, delicate and sharp, the biggest one narrow and slightly longer than the adjacent ones. Inner marginal tooth with about 27 very delicate and long, slender cusps, outer marginal tooth with about 30 delicate sharp cusps.

**Soft parts morphology and anatomy**: Head and mantle pigmented intensely black, ctenidium present.

**Female reproductive organs**: similar to the ones described by Odabaşı et al. [67] for *Pseudorientalia kanari*, with big and bulky bursa copulatrix and two receptacula seminis, the distal one small, nearly rudimentary, the proximal one big, thread-like. The female reproductive organs practically identical with the ones in *Arslania kanari*, only the oviduct accessory gland complex shorter and broader than in that species.

**Penis** (Figure 8K,L) gradually tapering with no outgrowths, moderately thick at its base, intensely black or brown pigmented from its middle to the tip, vas deferens visible inside unpigmented proximal part of the penis.

**Differential diagnosis**: apart from 2.5% divergence in the COI, these two species occurring in sympatry in the same spring, differ markedly in their shells and radulae. In *Arslania kybele* sp. nov. the shell is trochoid, neither conic nor oval as in *A. kanari*, shell much thicker-walled than in that species, cusps on all the teeth of the radula much longer and narrow than in *A. kanari*. Female reproductive organs and penes similar as in *A. kanari*. The MDCs in comparison with *Arslania kanari* sequences: binary: 47(A), 65(A), 89(C), 143(A), 194(A), 197(A), 242(A), 302(C), 350(A).

**Derivation of name**: The specific epithet “*kybele*” honours Kybele (Cybele), the ancient mother goddess of Phrygia and one of the principal deities of Anatolia, reflecting the historical landscape in which this species occurs. In classical iconography, Kybele is commonly depicted accompanied by two lions, a symbolic motif that also harmonizes with the generic name *Arslania* gen. nov., dedicated to Dr. Naime Arslan.

**Distribution and habitat**: Known from its type locality only.

**Caspiinae B. Dybowski, 1913**

**Genus *Erosiconcha* Delicado et Gürlek, 2021**

***Erosiconcha geldiayana* (Schütt et Bilgin, 1970)**

GenBank sequence numbers: QB223165

Locality 1 (Table 1, Figure 1).

The shell (Figure 5I), radula (Figure 6C) with two basal cusps on the central tooth, female reproductive organs and penis (Figure 8H) are similar to the ones described by Schütt & Bilgin [74] and Delicado & Gürlek [4].

**Genus *Graecoanatolica* Radoman, 1973**

***Graecoanatolica* cf. *tenuis* Radoman, 1973**

GenBank sequence numbers: QB223166–QB223168

Localities 2 and 3

**Shell**: (Figure 5J–L) up to 2.44 mm high, ovate-conical or conic, moderately broad (Figure 5H,I) or slender (Figure 5J), with spire from relatively low to high, with about 4^3^/_4–_5^1^/_4_ whorls, the spire’s height about 27–47% of the shell height, body whorl not massive. Teleoconch whorls moderately convex, evenly rounded, growing regularly in diameter. Aperture rather narrow, ovoid with angle at its upper part, outer lip simple, parietal lip complete and thin, umbilicus slit-like. Teleoconch moderately thick-walled, its wall translucent, with delicate growth lines, periostracum yellowish. Operculum smooth on both surfaces.

**Radula** (Figure 6D): central tooth with lateral angles highly expanded, ventral process well excavated, median cusp triangular and massive, adjacent cusps on the cutting edge short (less than half of the central cusp length), sharp, well separated from each other, two basal cusps on each side, rather big, located relatively low on lateral angles of central tooth. Central tooth formula:

$$\frac{5-1-5}{2-2}$$

Lateral tooth following formula: 4 − 1 − 5, cusps bigger than on the central tooth, broad and massive, the biggest one less than twice longer and more than twice broader than the adjacent ones. Inner marginal tooth with 19–27 long and slender cusps, outer marginal tooth with about 12 delicate sharp cusps.

**Soft parts morphology and anatomy**: Head and mantle intensely pigmented black.

**Female reproductive organs** (Figure 7H,I) similar to the ones described and figured by Radoman [62] for *G. vegorriticola*, only the pallial glands complex (albuminoid and nidamental gland) much shorter. No bursa copulatrix, S-shaped loop of oviduct broad and massive. Big, bulky and spherical, distal (at the position of rs_1_) receptaculum seminis.

**Penis** (Figure 8I) similar to the one described and figured by Radoman [62] for *G. vegorriticola*, gradually tapering, bent, its base moderately broad, delicately marked broad outgrowth proximally on the left side, vas deferens visible inside the penis.

**Remarks**: As visible in Figure 5J, the shell of this taxon is very variable. Thus, the description and photographs of Radoman [62,71] are not useful for species identification. The study of Kebapçi et al. [6] is also not very useful in this case. No anatomical data have been published. The female reproductive organs and penis confirm the genus assignment to *Graecoanatilica*. Our locality is rather close to the type locality (a big spring in the Gemiş village, about 13 km from Çardak, near the south bank of Lake Aci Gölü, Türkiye), similar to the one of Delicado et al. [9] (Türkiye: Lake Eğirdir). Our sequence is identical with one of those published by [9]. However, that species determination was not confirmed with anatomy [75]. Thus, the only solution is to classify this taxon as *Graecoanatolica* cf. *tenuis*.

Radoman [62] homologised this big, bulky and spherical sperm-containing structure with the distal receptaculum seminis, recording also a vestigial bursa copulatrix (in a form of “poor stump”)at the site where the bursa copulatrix duct opening should be located. Perhaps his interpretation of homology is justified. However, as reported, e.g., by [76] in some caenogastropod, there are structures of mixed function of the bursa copulatrix and receptaculum seminis. This can be checked only with histology: in such cases, some of the spermatozoans should be placed with their heads embedded in the epithelium of the structure which feeds them, and some other ones are free. Considering these functions, such a big sac rather must function as a bursa.

## 4. Discussion

Cytochrome c oxidase subunit I (COI) is widely used for species distinction and phylogeny reconstruction among rather closely related taxa. On the other hand, its application for reconstruction of deeper nodes is restricted, since saturation makes it hardly possible to infer older history. This may explain why our tree does not confirm the monophyly of the subfamily Horatiinae. On the other hand, the clades representing the subfamily Caspiinae and *Pseudorientalia* are well supported. Within the clade of *Pseudorientalia*, apart from the type species, the snails *Pseudorientalia homeriana* sp. nov. and *P. pythagorasiana* sp. nov. from the Chios and Samos Islands, respectively, are grouped both as distinct species. Unexpectedly, “*Tefennia*” *tefennica* clearly belongs to this clade. The genus, described by Schütt & Yıldırım [64] and later studied molecularly and anatomically by Çağlan et al. [59], has a shell with a characteristically sinuated outer lip, although this sinuation is not always present. The radula is not different from the one typical of *Pseudorientalia* [59], and the penis has a characteristic finger-like outgrowth on its left side, but a similar one can be found in some specimens of *Pseudorientalia natolica*. The big bursa copulatrix in “*Tefennia*” has no duct that is typical of the other *Pseudorientalia*, and no distal receptaculum seminis in *Tefennia* has been found so far [59,64]. However, a rudimentary distal receptaculum cannot be excluded in “*Tefennia*”, since the animal is minute, and the examined specimens [59] are not fixed well enough. The bursa and receptacula are prone to not only genetic but also physiological wide variation within a species [11]. A dwarf distal receptaculum is characteristic of *Pseudorientalia homeriana* sp. nov. as well. Thus, there is no strong morphological evidence contradicting the assignment of “*Tefennia*” *tefennica* to the genus *Pseudorientalia*, as clearly suggested by our molecular data, and the genus *Tefennia* Schütt et Yıldırım, 2003, becomes no longer valid, as a junior synonym of *Pseudorientalia* Radoman, 1983. The nuclear loci confirm close relationships of “*Tefennia*” with *Pseudorientalia natolica* (Supplementary Figure S2). Contrary to a species, a genus is not a really an existing entity but only a category. Thus, there are no strict rules defining a species, especially in such a poorly known group of animals. All the truncatelloid genera are still temporary. It has to be noted in this case that considering *Tefennia* a distinct genus would result in the rejection of the monophyly of *Pseudorientalia*, a well-supported (bootstrap 92) clade in our tree.

Unexpectedly, “*Pseudorientalia*”, recorded [3] from the Lesvos Island, belongs to a well-supported clade, which we have described as a new genus, *Sapphonia* gen. nov., outside of *Pseudorientalia* and close to *Grossuana* and *Daphniola*, still within the Horatiinae. Considering its placement in the molecular tree, it is together with *Grossuana* Radoman, 1983, and *Daphniola* Radoman, 1973, but molecularly equally distant from each of these genera than the latter between each other. It has a valvatoid shell, a dwarf distal receptaculum seminis and a penis with a much bigger outgrowth on its left side [68] that clearly distinguishes *Daphniola* from *Sapphonia* gen. nov. The shell in *Grossuana* is similar, but the bursa copulatrix in *Grossuana* is much smaller, the proximal receptaculum is bigger, and distally there is a rather big, bi-lobed outgrowth on the left side [51] that is not present in *Sapphonia* gen. nov. Thus, both the molecular and morphological data support genus-level distinctness of *Sapphonia* gen. nov. Both the penes and female reproductive organs of *Sapphonia* gen. nov. and *Pseudorientalia* are similar, despite their not-too-close phylogenetic relationships. Similar slight differences in morphology were observed [61] between the phylogenetically distant *Grossuana* and *Radomaniola* Szarowska, 2006. As stressed (e.g., [11]), the morphology of the soft parts in the Truncatelloidea, reproductive organs included, is miniaturized, simplified, physiologically variable and controlled by environmental factors causing homoplasies. This applies to not only a species but also at higher levels.

Another unexpected result is the molecularly inferred status of “*Pseudorientalia*” *kanari*. The simple, gradually tapering penis, the bursa copulatrix being expanded over the albuminoid gland, and two receptacula seminis, coupled with a conic or ovate-conic shell, resulted in its assignment to the genus *Pseudorientalia*. However, also in this case, the morphology was misleading. Molecularly, this taxon is far from *Pseudorientalia* and cannot be assigned to any genus molecularly studied so far. Its sister taxa are *Martensamnicola* from Central Asia and *Iranobelgrandia* from Iran, which may reflect the Asiatic element of the Turkish malacofauna. It is noteworthy that in the same locality, in sympatry, two distinct species belonging to the same new genus *Arslania* gen. nov. have been found.

The Truncadelloidea are a diverse group, and there is no threshold value for species distinction (e.g., [11,53,76,77]). For the most often used COI it ranges, for p or K2P distances, from 1.4 to 4% depending on the group, although most often it does not exceed 2%. Thus, a 2.5% of divergence in COI rather surpasses the threshold value for species distinction in these snails. Mitochondrial DNA does not always reflect species evolution; thus, for definitive decisions about species distinctness some nuclear loci should be also considered. In the *Arslania* gen. nov. case, however, this molecular COI divergence is coupled with different shell morphologies (conic or ovate-conic vs trochoid) and, more importantly, by the distinct differences of the shell-wall thickness. The shell thickness depends on several habitat factors, like predation (e.g., [78,79,80]), but within the same small spring it may be determined only by genetic factors. The radulae also were different in these species. Thus, in this case the mtDNA seems to reflect the species, not molecule evolution. We have to stress that despite the wide variability in shell characters, markedly different shell-wall thicknesses (at the same locality and at the same time) coupled with differences in the cusps in the radula, are rather unusual between closely related species of the Truncatelloidea. We have confirmed these conclusions by differences in nuclear histone H3 sequences between these two *Arslania* species (Supplementary Figure S2).

As discussed above we have observed similar morphologies, not only of the shell but also of the reproductive organs, between distantly related taxa. However, there is the other side of the coin in the truncatelloidean systematics: a species-level revision of *Graecoanatolica* [6], based entirely on the shell characters, as in the revision formerly published [6], would result in the identification of at least two such “morphospecies” (with morphology restricted to the shell alone) in our specimens representing this genus. Molecularly, however, they clearly belong to one species; thus, the shell characters alone cannot be used to distinguish a species. Such conclusions are already known from the time of Roszkowski [81] but were later confirmed by Hubendick [82], Giusti & Pezzoli [83], Muus [84,85], and Wilke & Falniowski [86]. Later, the morphology and anatomy of the soft parts also proved to be restricted in species distinction [12,31,87], and the “lock-and-key” mechanism does not always work [88]. As concerns *Graecoanatolica* cf. *tenuis*, it was collected from two localities: 2 and 3. Locality 2—Akpınar Spring, Akpınar Village, Başmakçı, Afyonkarahisar, Türkiye—is very close to the type localities of *G. tenuis* (5.5 km) and *G. conica* (10.3 km). The remaining known *Graecanatolica* localities in Türkiye are considerably farther away, for example, *G. dinarica* (33 km), *G. brevis* (30 km), and *G. lacustristurca* and *G. kocapinarica* (85 km). Locality 3—Kayalıköy Village, Karamanlı, Burdur—is geographically isolated from all other known *Graecanatolica* localities in Türkiye. The nearest type locality is that of *G. brevis* (32 km away), whereas the type localities of the other species are more distant, including *G. tenuis* (55 km), *G. conica* (61 km), *G. dinarica* (87 km), and *G. lacustristurca* (102 km). It has to be noted, however, that the shell in our Figure 5J (but not the other two from this locality) rather resembles *G. lacustriturca* Radoman, 1973, than *G. tenuis*, but this illustrates how variable the shell in *Graecoanatolica* is; thus, we have decided to follow Delicado et al. [9], as well as the geographic distribution of the *Graecanatolica* nominal species, with our locality being close to type one in the species assignment of these specimens.

Apart from the commonly found insufficiency of the morphological characters in the species distinction, our study revealed the limitations of morphological traits in the higher-level systematics. A very similar gross anatomy, simple penes and practically the same female reproductive organs unified three distinct genera: *Pseudorientalia, Sapphonia*, and *Arslania*. The sister genera of each of these three present strikingly different morphologies; thus, there is an example of homoplasy, perhaps parallelism. Such parallelisms, including even the gain and loss of complicated structures, like tubular penial glands, have already been reported [89].

## 5. Conclusions

Our results show that morphology, particularly shell characters, can be highly misleading in hydrobiid systematics when used without molecular data. Phylogenetic analyses revealed two new *Pseudorientalia* species, supported the synonymization of *Tefennia* with *Pseudorientalia*, and led to the recognition of two new genera and one more previously unrecognized species. The study emphasizes the value of integrative taxonomy for uncovering hidden biodiversity and reconstructing evolutionary relationships in freshwater Hydrobiidae. It is also noteworthy that apart from the evidence of the distinctness of four previously undescribed species, the molecular data have demonstrated that within the previously morphologically defined genus *Pseudorientalia*, two distinct genera have been distinguished, *Sapphonia* and *Arslania*, both phylogenetically far from *Pseudorientalia*. The sister taxa of these two genera are characterised by apparently distinct morphologies. On the other hand, in *Graecoanatolica*, shells characteristic of at least two easily distinguishable “species” evidently belonged to the same taxon.

## Figures and Tables

**Table 1:** Sampling localities with geographical coordinates. See also Figure 1 and Figure 2.

Id	Site	Coordinates	Sequence ID
*Erosiconcha geldiayana* (Schütt & Bilgin, 1970)			
Site 1	A big spring flowing into near Yarışlı Gölü, between the Kocapınar Köyü and Sazak Köyü, Burdur, Türkiye, 929 m a.s.l.	37°32′43.1″ N 29°56′37.3” E	3M72
*Graecoanatolica* cf. *tenuis* Radoman, 1973			
Site 2	A big spring (Akpınar Kaynağı-Spring) flowing into Acı Göl., Afyonkarahisar, Başmakçı, Akpınar Köyü, Türkiye, 853 m a.s.l.	37°49′00.5″ N 29°55′33.6″ E	3M76
Site 3	Kocapınar Spring, Burdur—Karamanlı—near Kayalı Köyü, Türkiye, 1005 m a.s.l.	37°18′23.0″ N 29°55′56.3″ E	3M78, 3M79
*Arslania kanari* (D. A. Odabaşı, 2024) comb nova *Arslania kybele* sp. nov.			
Site 5	Göksu Spring, Göktepe village, Sivrihisar district, southeast of Eskişehir province, Türkiye, 845 m a.s.l.	39°09′01.4″ N 31°36′49.32″ E	3O78–3O85
*Pseudorientalia homeriana* sp. nov.			
Site CH01	Manna Nerou spring, near Ghiosonas, Chios Island, Greece, 30 m a.s.l.	38°33′28.7″ N 26°04′50.2″ E	KJ920492, KJ920493, KJ920497
*Pseudorientalia natolica* (Küster, 1853)			
Site 4	Pınarbaşı spring in Gedelek village, Bursa, Türkiye, 189 m a.s.l. Turkey (located near Gemlik, just north of Bursa); type locality	40°27′07.2″ N 29°15′38.9″ E	3i80, 3i81
*Pseudorientalia pythagorasiana* sp. nov.			
Site S02	Despoti Fountain between Koutsi and Pirgos, Samos Island, Greece, 490 m a.s.l.	37°42′59.0″ N 26°46′51.0″ E	KJ920473–KJ920477
*Pseudorientalia tefennica* (Schütt et Yıldırım, 2003) comb. nova			
Site 1	A big spring flowing into near Yarışlı Gölü, between the Kocapınar Köyü and Sazak Köyü, Burdur, Türkiye, 929 m a.s.l.	37°32′43.1″ N 29°56′37.3″ E	3M73
*Sapphonia lyrica* sp. nov.			
Site L01	A spring near Klió, Epetimnos Mountain, Lesvos Island, Greece, 208 m a.s.l.	39°21′30.5″ N 26°18′39.7″ E	KJ920478–KJ920484
Site L02	A spring SSW of Argennos Epetimnos Mountain, Lesvos Island, Greece, 362 m a.s.l.	39°21′41.1″ N 26°16′14.7″ E	KJ920485, KJ920489

## Data Availability

The sequences were deposited in GenBank (accession numbers: QB223162–-QB223176, QB209838–QB20985, QB212630–QB212644, QB212645–QB212659) at https://www.ncbi.nlm.nih.gov (accessed on 26 October 2026).

## Supplementary Materials

The following supporting information can be downloaded at https://www.mdpi.com/article/10.3390/biology15211963/s1, Supplementary Figure S1: Shells of the Aegean species (after Szarowska et al. [2,3]): A–C—*Pseudorientalia homeriana* sp. nov., Chios Island; D,G—*Pseudorientalia pythagorasiana* sp. nov.; Samos Island; H–N—*Sapphonia lyrica* sp. nov., Lesvos Island. Bar equals 1 mm. Supplementary Figure S2. Maximum Likelihood tree based on the 18S, 28S andH3 genes. Bootstrap supports (>70%) are shown at the nodes. Newly obtained sequences are highlighted in bold. Supplementary File S1: Fasta file used in the MDC analysis.
